# Galectin-1 and galectin-3 expression in equine mesenchymal stromal cells (MSCs), synovial fibroblasts and chondrocytes, and the effect of inflammation on MSC motility

**DOI:** 10.1186/s13287-017-0691-2

**Published:** 2017-11-02

**Authors:** Heidi L. Reesink, Ryan M. Sutton, Carolyn R. Shurer, Ryan P. Peterson, Julie S. Tan, Jin Su, Matthew J. Paszek, Alan J. Nixon

**Affiliations:** 1 Department of Clinical Sciences, College of Veterinary Medicine, Cornell University, Ithaca, NY 14853 USA; 2 Robert Frederick Smith School of Chemical and Biomolecular Engineering, Cornell University, Ithaca, NY 14853 USA

**Keywords:** Osteoarthritis, Adhesion, Migration, Horse, Stem cell, IL-1β, TNF-α

## Abstract

**Background:**

Mesenchymal stromal cells (MSCs) can be used intra-articularly to quell inflammation and promote cartilage healing; however, mechanisms by which MSCs mitigate joint disease remain poorly understood. Galectins, a family of β-galactoside binding proteins, regulate inflammation, adhesion and cell migration in diverse cell types. Galectin-1 and galectin-3 are proposed to be important intra-articular modulators of inflammation in both osteoarthritis and rheumatoid arthritis. Here, we asked whether equine bone marrow-derived MSCs (BMSCs) express higher levels of galectin-1 and -3 relative to synovial fibroblasts and chondrocytes and if an inflammatory environment affects BMSC galectin expression and motility.

**Methods:**

Equine galectin-1 and -3 gene expression was quantified using qRT-PCR in cultured BMSCs, synoviocytes and articular chondrocytes, in addition to synovial membrane and articular cartilage tissues. Galectin gene expression, protein expression, and protein secretion were measured in equine BMSCs following exposure to inflammatory cytokines (IL-1β 5 and 10 ng/mL, TNF-α 25 and 50 ng/mL, or LPS 0.1, 1, 10 and 50 μg/mL). BMSC focal adhesion formation was assessed using confocal microscopy, and BMSC motility was quantified in the presence of inflammatory cytokines (IL-1β or TNF-α) and the pan-galectin inhibitor β-lactose (100 and 200 mM).

**Results:**

Equine BMSCs expressed 3-fold higher galectin-1 mRNA levels as compared to cultured synovial fibroblasts (*p* = 0.0005) and 30-fold higher galectin-1 (*p* < 0.0001) relative to cultured chondrocytes. BMSC galectin-1 mRNA expression was significantly increased as compared to carpal synovial membrane and articular cartilage tissues (*p* < 0.0001). IL-1β and TNF-α treatments decreased BMSC galectin gene expression and impaired BMSC motility in dose-dependent fashion but did not alter galectin protein expression. β-lactose abrogated BMSC focal adhesion formation and inhibited BMSC motility.

**Conclusions:**

Equine BMSCs constitutively express high levels of galectin-1 mRNA relative to other articular cell types, suggesting a possible mechanism for their intra-articular immunomodulatory properties. BMSC galectin expression and motility are impaired in an inflammatory environment, which may limit tissue repair properties following intra-articular administration. β-lactose-mediated galectin inhibition also impaired BMSC adhesion and motility. Further investigation into the effects of joint inflammation on BMSC function and the potential therapeutic effects of BMSC galectin expression in OA is warranted.

**Electronic supplementary material:**

The online version of this article (doi:10.1186/s13287-017-0691-2) contains supplementary material, which is available to authorized users.

## Background

Joint trauma, inflammation and the subsequent development of osteoarthritis (OA) are common in both humans and horses. Joint inflammation is precipitated by the production of catabolic cytokines, such as interleukin-1 beta (IL-1β) and tumor necrosis factor alpha (TNF-α), in addition to immune cell infiltration. Mesenchymal stem cells (MSCs) were initially appealing as a cell source for the repair of articular cartilage and other musculoskeletal injuries due to their multilineage potential [1]. However, low rates of long-term survival, engraftment and differentiation in musculoskeletal tissues [2, 3] led to a failure to observe many of these properties. Thus, the paradigm for intra-articular MSC-based therapies has shifted from MSCs as tissue reparative cells to MSCs as immunomodulatory cells that modify the microenvironment to encourage tissue repair by endogenous tissue progenitor cells [4, 5]. MSCs have been used intra-articularly to decrease joint inflammation and promote cartilage healing in both experimental animal models of OA [3, 6, 7] and in human clinical trials [8, 9]; however, the mechanisms by which MSCs perform these actions are poorly understood.

Various secreted molecules have been implicated in the anti-inflammatory and regenerative properties of stem cells, including prostaglandin E_2_ (PGE_2_) [4, 10], indoleamine 2,3-dioxygenase (IDO) [11], interleukin-10 (IL-10) [4], tumor necrosis factor-inducible gene 6 (TSG-6) [12] and interleukin-1 receptor antagonist (IL-1Ra) [13]. Recently, galectin-1 and galectin-3 have been proposed as potent mediators of MSC immunomodulatory properties [14–18] and MSC motility [19]. Human MSCs constitutively express galectins-1, -3 and -8 [15, 20], with abundant expression of galectins-1 and -3 in human umbilical cord blood-derived MSCs [16]. Both galectin-1 [17, 21, 22] and galectin-3 [14–17, 21, 23] appear to play a significant role in the ability of human MSCs to downregulate immune responses. Furthermore, galectin-1 has been shown to induce chondrogenic differentiation of MSCs from the bone marrow of rheumatoid arthritis patients [24]. However, galectin expression has not been previously investigated in equine MSCs.

Galectins comprise a family of more than 15 β-galactoside binding proteins [25, 26]. Galectin-1 possesses immunosuppressive and anti-inflammatory effects in many chronic inflammatory and autoimmune disorders, including graft-versus-host disease [27] and systemic lupus erythematosus [28]. Decreased galectin-1 and increased galectin-3 synovial fluid concentrations have been reported in human patients with OA [29], juvenile idiopathic arthritis [30, 31] and rheumatoid arthritis [32]. Galectin-3 has recently been shown to enhance cartilage boundary lubrication via interactions with lubricin/proteoglycan 4 [33]; however, galectin-3 gene expression is increased in OA cartilage as compared to normal cartilage [34]. Increased galectin-3 histochemical staining has also been observed in the synovial membrane and articular cartilage of arthritis patients [31, 35, 36]. In a collagen-induced arthritis model, gal-1 null (-/-) mice demonstrated an accelerated disease onset and more severe arthritis as compared to wild-type mice [37]. Both galectin-1 gene therapy and recombinant galectin-1 intra-articular administration abrogated clinical and histopathological signs of collagen-induced arthritis in a murine model [38], suggesting that galectin-1 may be a possible therapeutic option for inflammatory arthritis. Beyond their role as immunomodulatory proteins, galectins also have diverse effects on cell adhesion, chemotaxis [39, 40] and migration [26].

Interestingly, many of the protagonists of MSC effects, including VEGF, IDO, PGE_2_ and galectin-9, are increased following exposure to inflammatory stimuli, including interferon gamma (IFN-ϒ), TNF-α and lipopolysaccharide (LPS) [41, 42]. TNF-α, one of the major inflammatory cytokines in OA, has been shown to enhance the regenerative potential and anti-inflammatory response in MSCs [41]. Priming of equine MSCs with TNF-α and IFN-ϒ significantly upregulated COX-2, iNOS, IDO and IL-6 gene expression and adhesion-related genes, while downregulating migration-related genes [43]. Whereas exposure to high concentrations (50 ng/mL) of TNF-α and IFN-ϒ negatively affected equine bone marrow-derived MSC (BMSC) viability and differentiation, lower concentrations (5 ng/mL) did not demonstrate adverse effects [44]. To our knowledge, it is unknown how pro-inflammatory cytokines impact galectin expression, adhesion and motility in equine BMSCs. MSC adhesion to articular cartilage is critical for cartilage repair, and priming of MSCs with hyaluronan has been demonstrated to increase MSC attachment to cartilage [45]. Following joint injury, MSCs must mobilize to injured tissues. Interestingly, anterior cruciate ligament rupture in a rat model induced migration of intravenously injected MSC to the synovium of the injured joint [46]. Although no changes in serum cytokines were observed between rupture and control groups in this study, synovial fluid cytokines were not evaluated.

Given the significance of galectin-1 and -3 in articular inflammation, we asked how equine BMSC galectin-1 and -3 expression compares to other articular tissue-derived cells and whether inflammatory cytokine priming would alter galectin-1 and galectin-3 expression in BMSCs. Second, we investigated the role of the inflammatory cytokines IL-1β and TNF-α and the pan-galectin inhibitor β-lactose on BMSC motility. We hypothesized that equine BMSCs would express high levels of galectin-1 and -3, and that galectin-1 may account for some beneficial properties of intra-articular BMSC administration. Therefore, the overall objectives of our study were to: (1) quantify galectin-1 and -3 gene expression in equine BMSCs, synoviocytes and chondrocytes, and (2) evaluate the effect of inflammatory cytokines on BMSC galectin expression, protein expression, protein secretion and BMSC motility.

## Methods

All animal and tissue harvesting protocols were approved by Cornell University’s Institutional Animal Care and Use Committee (Protocol Number: 2011-0027).

### Cell isolation and culture

Bone marrow was collected from the sternebrae of 31 standing, sedated Quarter Horse, Warmblood and Thoroughbred horses (*n* = 10 female, 2 intact male and 19 castrated male) ranging in age from 3 to 12 years old for primary BMSC isolation as previously described [47]. Briefly, bone marrow biopsy needles (Jamshidi, VWR Scientific, Bridgeport, NJ, USA) were used to aspirate 60–180 mL of sternebral bone marrow into one to three 60 mL syringes containing heparin (APP Pharmaceuticals, LLC, Schaumburg, IL, USA) at a final concentration of 1,000 units/mL. BMSCs were isolated via selective tissue culture plastic adherence after plating bone marrow 1:1 in Dulbecco’s modified Eagles’ media; 1,000 mg/L glucose (Gibco-Life Technologies, Grand Island, NY, USA) supplemented with 1 ng/mL bFGF (Gibco, Invitrogen, Camarillo, CA, USA), 25 mM HEPES (Gibco-Life Technologies, Grand Island, NY, USA), 100 units/mL penicillin-streptomycin, and 10% fetal calf serum. Non-adherent cells were removed via media changes every other day. Following colony formation, adherent cells were passaged and replated at 10–12,000 cells/cm^2^. Cells were thawed following cryopreservation, cultured for 72 h, collected in lysis buffer and frozen at ˗80 °C for subsequent RNA isolation for constitutive galectin gene expression analysis. Bone marrow was collected from the sternebrae of an additional six standing, sedated Thoroughbred-type horses (n = 2 female and 4 castrated male) ranging in age from 1 to 8 years old and culture expanded as described above for use in cytokine gene and protein expression, confocal imaging and migration experiments. All cells were stored cryopreserved prior to experiments.

### Differentiation assay

Adipogenic, osteogenic and chondrogenic differentiation potential of primary BMSC lines was evaluated using standard protocols following induction with StemPro® differentiation kits (Thermo Fisher Scientific, Waltham, MA, USA). Adipogenic and osteogenic differentiation were induced in confluent monolayer cultures, and chondrogenic differentiation was induced in 5 μL cell pellets. Differentiated cells and non-induced controls were stained with Oil Red O, Alizarin Red, or Toluidine Blue between 7 and 14 days and photographed with a × 20, 0.7 NA objective using an Olympus IX73 microscope equipped with an Olympus DP80 CCD camera (Olympus, Tokyo, Japan).

Synovial membrane (*n* = 27) and articular cartilage (*n* = 16) tissues were aseptically harvested from the shoulder, stifle, carpal and fetlock joints of Thoroughbred, Warmblood and Quarter Horse cadavers (*n* = 9 female, 6 intact male and 12 castrated male) ranging in age from 1 to 8 years old immediately post-euthanasia. All horses were healthy without gross signs of OA at dissection. Cells were isolated, pooled and culture expanded as previously described for synoviocytes [48] and chondrocytes [49]. Briefly, synovial lining was digested in 0.15% collagenase (Worthington Biochemical, Lakewood, NJ, USA) and 0.015% DNAseI (Roche, Indianapolis, IN, USA) for 3 h at 37 °C, followed by filtration and centrifugation at 250 × *g* for 10 minutes. Synoviocytes were cultured in Dulbecco’s modified Eagles’ media; 4500 mg/L glucose (Gibco-Life Technologies, Grand Island, NY, USA) supplemented with 25 mM HEPES, 100 units/mL penicillin-streptomycin, and 10% fetal calf serum. Articular cartilage was digested in 0.075% collagenase overnight at 37 °C, followed by filtration and centrifugation at 250 × *g* for 10 minutes. Chondrocytes were cultured in Ham’s F12 medium (Corning Inc., Corning, NY, USA) supplemented with 50 μg/mL ascorbic acid, 30 μg/mL α-ketoglutarate, 300 μg/mL L-glutamine, 25 mM HEPES, 100 units/mL penicillin-streptomycin and 10% fetal calf serum.

### Equine galectin gene expression analysis

For constitutive galectin expression, passage 1 to 3 equine BMSCs (*n* = 31), synovial fibroblasts (*n* = 27) and chondrocytes (*n* = 16) were cultured for 72 h after thawing in appropriate growth media as discussed above. Cells were collected in lysis buffer and frozen at ˗80 °C for subsequent RNA isolation using a purification kit (5 Prime Inc., Gaithersburg, MD, USA). Synovial membrane and cartilage tissue obtained from healthy equine carpal joints at the time of surgery or euthanasia (*n* = 23) from a previous study [50] that had been stored at ˗80 °C was thawed for RNA isolation and purification using the same purification kit. RNA purity and concentration were assessed using UV microspectrophotometry (NanoDrop 2000, Thermo Fisher Scientific, Waltham, MA, USA). Gene expression was quantified through the use of quantitative real-time polymerase chain reaction (qRT-PCR) using the ABI PRISM 7900 sequence detection system (Applied Biosystems, Foster City, CA, USA), with all samples analyzed in duplicate using primers and a dual-labeled fluorescent probe (6-FAM™ as the 5’ reporter label and Iowa Black® FQ as the 3’ quenching label). Primers and probes were generated using the equine galectin-1 and -3 sequences as described above and were designed using Primer Express software (Primer Express v2.0b8, Foster City, CA, USA):

- (Galectin-1 Fwd: 5’- CAAGGCAGACCTGACCATCA -‘3,
- Galectin-1 Rev: 5’- TCACGGCCTCCAGGTTGA -3’,
- Galectin-1 Probe: 5’-/56-FAM/CTGCCGGAT/ZEN/GGCTACTCGTTCAAGTTC/3IABkFQ/-3’,
- Galectin-3 Fwd: 5’- TAAATTTCAACAGAGGGCATGATG -3’,
- Galectin-3 Rev: 5’- CAATGACTCTCCTGTTGTTCTCGTT -3’
- Galectin-3 Probe: 5’-/56-FAM/TGCCTTCCA/ZEN/CTTTAACCCGCGCTT/3IABkFQ/-3’).

Total copy number of mRNA was determined from a validated standard curve using serial dilutions of *E. coli*-expressed equine galectin-1 and -3 as standards for absolute quantitation, and copy number was normalized to the housekeeping gene 18S.

### Cytokine treatments

For inflammatory cytokine treatments, passage 3 BMSCs (*n* = 3) were plated in duplicate in 24-well plates at a concentration of 2 × 10^4^ cells/cm^2^. BMSCs remained in serum-containing MSC growth media for 24 h prior to cytokine treatments. Serum-containing media was replaced with serum-free Opti-MEM (Invitrogen, Grand Island, NY, USA) 4 h prior to stimulation. BMSCs were stimulated with recombinant equine IL-1β (IBI Scientific, Peosta, IA, USA) at 5 ng/mL and 10 ng/mL, recombinant equine TNF-α (IBI Scientific, Peosta, IA, USA) at 25 ng/ml and 50 ng/ml or LPS from *E. coli* 055:B5 (Sigma-Aldrich, St. Louis, MO, USA) at 0.1 μg/mL, 1 μg/mL, 10 μg/mL and 50 μg/mL. BMSCs remained in serum-free Opti-MEM as the control condition. Media supernatants were collected at 4, 8, 20 and 30 h post-treatment, frozen and stored at ˗80 °C for galectin quantification via custom ELISA. Cells were lysed at 4, 8, 20 and 30 h after treatment for RNA isolation, and gene expression was determined using qRT-PCR for galectin-1 and galectin-3 mRNA with 18S used as a housekeeping gene. In parallel, BMSCs were lysed at 4, 8, 20 and 30 h after treatment with ice-cold RIPA buffer containing protease inhibitors. Cell lysates were stored at -80 °C for immunoblotting analysis.

### Equine galectin protein expression after cytokine stimulation

Custom ELISAs were developed for the detection of equine galectin-1 and galectin-3 in BMSC media supernatants following cytokine stimulation. Equine galectin-1 (GenBank ID: KY264050) and galectin-3 (GenBank ID: KY264051) were cloned and sequenced from renal tissue obtained from a 19-year-old Thoroughbred cadaver mare as previously reported [33]. In order to assess antibody cross-reactivity and to establish equine-specific standards for galectin ELISAs, equine galectins-1 and -3 were recombinantly expressed and purified as described for human galectins-1, -3 and -3C [51]. All antibodies were validated to react against purified equine galectin-1 or galectin-3 using dot blots (Additional file 1). Briefly, for the custom competitive equine galectin-1 ELISA, 96-well high-binding plates (Corning Inc., Corning, NY, USA) were coated with 1 ug/mL of capture antibody (R&D Systems, Minneapolis, MN, USA; goat anti-mouse Gal-1 pAb, AF1245) in sodium carbonate buffer, pH 9.6 overnight at 4 °C. After rinsing 3× in 0.1% PBS-Tween, protein-free blocking buffer (Thermo Fisher Scientific, Rockford, IL, USA) was added for 1 h. Unlabeled recombinant equine galectin-1 standards (4000 to 15.63 ng/mL, plus 0 ng/mL) were diluted in a solution of 200 ng/mL biotinylated recombinant equine galectin-1 in 0.1% PBS-Tween. Supernatants from passage 3 BMSCs (*n* = 3) were collected in duplicate for all treatment conditions (control, IL-1β 5 and 10 ng/mL, TNF-α 25 and 50 ng/ml, and LPS 0.1 μg/mL, 1 μg/mL, 10 μg/mL and 50 μg/mL) at all time points (4, 8, 20 and 30 h). Supernatants were diluted 1:10 in 200 ng/mL biotinylated recombinant equine galectin-1 in 0.1% PBS-Tween. Blocking buffer was removed, and 100 μL of recombinant equine galectin-1 standards or samples were added to wells, covered, and incubated for 1 h at RT on a plate shaker. After rinsing 3× in 0.1% PBS-Tween, 100 μL of streptavidin HRP was added for 30 minutes, followed by 5× rinses in 0.1% PBS-Tween. TMB reagent (Thermo Fisher Scientific, Rockford, IL, USA) was added for 30 minutes, and the reaction was stopped with 1 N H_2_SO_4_. Absorbance was measured at 450 nm with 540 nm background subtraction, and all measurements were performed in duplicate.

For the equine galectin-3 sandwich ELISA, 96-well high-binding plates were coated with 2 ug/mL of capture antibody (Santa Cruz Biotechnology, Dallas, TX, USA; goat anti-human Gal-3 pAb, sc-19280) in sodium carbonate buffer, pH 9.6 overnight at 4 °C. After rinsing 3× in 0.1% PBS-Tween, protein-free blocking buffer was added for 1 h. Blocking buffer was removed, and serial dilutions of recombinant equine galectin-3 standards (400 ng/mL to1.56 ng/mL, plus 0 ng/mL) or 1:1 dilutions of BMSC supernatants in PBS + 2% BSA were incubated for 1 h. After rinsing 3× in 0.1% PBS-Tween, biotinylated detection antibody (R&D Systems Minneapolis, MN, USA; biotinylated goat anti-mouse Gal-3 pAb, BAF1197) was added at 200 ng/mL for 1 h. Plates were rinsed, and 100 μL of streptavidin HRP was added for 30 minutes, prior to 3× rinses in 0.1% PBS-Tween and addition of TMB reagent for 30 minutes. The TMB reaction was stopped with 1 N H_2_SO_4_, and absorbance was measured at 450 nm with 540 nm background subtraction. All measurements were performed in duplicate.

In order to measure intracellular and membrane-bound galectins after cytokine stimulation, cell lysates were thawed and heated at 95 °C in reducing SDS-PAGE loading buffer for 15 minutes. 10 μL of sample per well was loaded onto a 7.5% TGX gel (Bio-Rad, Hercules, CA, USA), and subjected to SDS-PAGE for 1 h at 100 V. Gels were transferred to PVDF membranes (EMD Millipore, Billerica, MA, USA), and immunoblotting was performed using antibodies against galectin-1 (R&D Systems, Minneapolis, MN, USA) goat anti-mouse Gal-1 pAb, AF1245), galectin-3 (Santa Cruz Biotechnology, Dallas, TX, USA; goat anti-human Gal-3 pAb, sc-19280) and actin (Santa Cruz Biotechnology, Dallas, TX, USA; goat anti-human actin pAb, sc-1615). Band intensities were quantified using NIH Fiji, with background subtraction, and galectin-1 and -3 band intensities were normalized to actin. Only monomeric galectins (approximately 17 kDa for galectin-1 and approximately 31 kDa for galectin-3) were quantified.

### BMSC nucleofection and confocal imaging

Passage 3 to 4 equine BMSCs (*n* = 3) were nucleofected with the fluorescent-protein tagged adhesion markers paxillin mEmerald and vinculin mApple using a human MSC nucleofector kit (Lonza, Basel, Switzerland). Cells were passaged at 48 h post-nucleofection and plated onto 12-well fibronectin-coated glass plates at a concentration of 2 × 10^3^ cells/cm^2^ in serum-free media (Dulbecco’s modified Eagles’ media, 1000 mg/L glucose; 1 ng/mL bFGF; 25 mM HEPES; 100 units/mL penicillin-streptomycin). BMSCs were plated in the presence and absence of 100 mM β-lactose (Santa Cruz Biotechnology Inc., Dallas, TX, USA). At 8 h post-plating, glass plates were fixed in 4% paraformaldehyde for 10 minutes, rinsed in PBS, followed by actin staining with phalloidin Alexa647 and nuclear staining with DAPI for 15 minutes, followed by several PBS rinses. Cells were imaged with a × 100, NA 1.3 objective on an Olympus IX83 microscope equipped with a Yokagawa X1 spinning disk confocal scanning unit and an Andor Ultra 897 EMCCD camera. Images were overlaid in Adobe Photoshop CS5 (Adobe Systems Inc., San Jose, CA, USA).

### Equine BMSC migration assays

Passage 3 to 4 equine BMSCs (*n* = 3) were plated onto 24-well tissue culture plates (Corning Inc., Corning, NY, USA) within silicone inserts containing a defined, 500 μm cell-free gap (Ibidi®, Martinsried, Germany) for migration assays. After 6 h, media was changed to either control media, media containing β-lactose (100 mM, 200 mM) or media supplemented with recombinant equine IL-1β (5 ng/mL, 10 ng/mL) or recombinant equine TNF-α (25 ng/ml 50 ng/ml). Twenty hours later, inserts were removed and media was replaced with control media or media containing β-lactose (100 mM, 200 mM). Phase contrast images were obtained at 0, 3, 8, 12, 24 and 48 hours following insert removal, using three images/well obtained with a × 10, NA 0.25 objective on an Olympus CK2 microscope with a Nikon Digital Sight DS-Fi1 CCD camera to image the entire cell-free gap. NIH Fiji was used to define the x,y coordinates of the leading edges of cells migrating across the cell-free region. Custom software (Python Software Foundation, Wilmington, DE, USA) was designed to measure the mean linear cell-free distance for each image, which was normalized to the cell-free distance at time zero for each treatment.

### Statistical analysis

For constitutive galectin gene expression, a one-way ANOVA with Tukey’s post hoc tests was performed on log-transformed gene expression data for galectin-1 and galectin-3 using statistical software (GraphPad Software Inc., La Jolla, CA, USA). Summary statistics were performed on the untransformed data, and significance was set at *p* < 0.05. Galectin-1 and galectin-3 gene expression after stimulation data were compared using a Friedman nonparametric test, matching data by primary cell line (GraphPad Software Inc., La Jolla, CA, USA). Post hoc comparisons between all treatment groups and the control group were made using the method described by Gibbons and Chakraborti (p. 459, equation 2.13) [52] with a Bonferroni correction for multiple comparisons and significance set at *p* < 0.05.

Galectin-1 and galectin-3 immunoblotting and ELISA protein expression after stimulation data were also compared using a Friedman nonparametric test, matching data by primary cell line (GraphPad Software Inc., La Jolla, CA, USA). Post hoc comparisons between all treatment groups and the control group were made using the method described by Gibbons and Chakraborti (p. 459, equation 2.13) [52] with a Bonferroni correction for multiple comparisons and significance set at *p* < 0.05. Migration experiments were performed using three BMSC primary cell lines, and all treatment conditions were applied in duplicate. The normalized mean cell-free distances were analyzed using statistical software (JMP 11.0, Cary, NC, USA) to perform linear regression model fitting. Parameters included treatment, time, and BMSC primary cell line. Tukey’s honest significant difference (HSD) post hoc tests were performed to compare the effects of time and BMSC primary cell line, with significance set at *p* < 0.05. Dunnett’s post hoc tests were performed to compare the effects of each treatment condition to the control condition, with significance set at *p* < 0.05.

## Results

### Differentiation assay

**Fig. 1:**
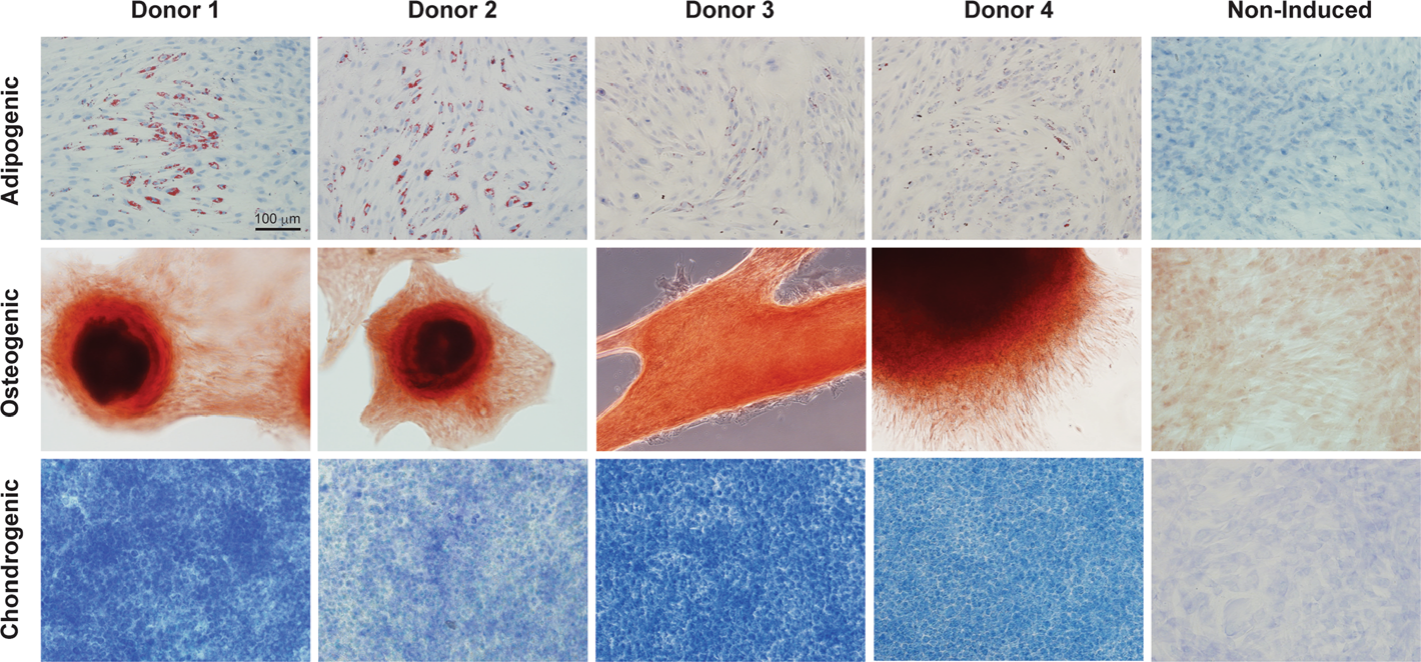
Tri-lineage differentiation of equine BMSCs. Representative microscopic images of Oil Red O, Alizarin Red, and Toluidine Blue staining to confirm adipogenic, osteogenic and chondrogenic differentiation of plastic adherent bone-marrow derived cell populations from four adult equine primary BMSC donors 7–14 days post-induction. Representative non-induced images from donor 1 are included for comparison. All images are obtained with a × 20, 0.7 NA objective

Tri-lineage differentiation capability was demonstrated in all primary BMSC lines used for cytokine stimulation experiments, confocal imaging experiments, and migration experiments (Fig. 1).

### BMSC galectin expression

**Fig. 2:**
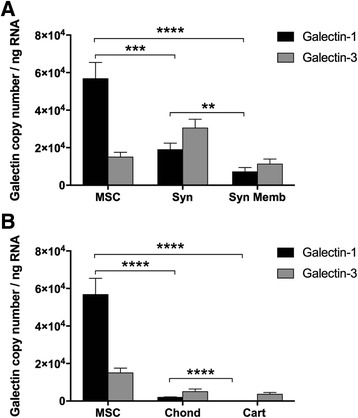
Constitutive galectin gene expression. Galectin-1 and galectin-3 transcription levels (copy number/ng RNA) from (**a**) equine BMSCs (*MSC*, n = 31), cultured synoviocytes (*Syn*, n = 27) and freshly harvested carpal synovial membrane tissue (*Syn Memb*, n = 23) and (**b**) equine BMSCs (*MSC*, n = 31), cultured chondrocytes (*Chond*, n = 16) and freshly harvested carpal articular cartilage (*Cart*, n = 10). Data are presented as mean ± SE. Statistical analysis is performed on log-transformed data (**p < 0.01, ***p < 0.001, ****p < 0.0001, Tukey’s post hoc tests)

BMSCs expressed approximately 3-fold higher galectin-1 as compared to cultured synoviocytes (*p* = 0.001) and 30-fold higher galectin-1 (*p* < 0.0001) relative to cultured articular chondrocytes (Fig. 2). Galectin-1 mRNA expression was 8-fold higher in BMSCs as compared to freshly isolated synovial membrane tissue (*p* < 0.0001) and more than 600-fold higher in BMSCs than articular cartilage (*p* < 0.0001). BMSC galectin-3 expression was not significantly different from cultured synoviocytes, cultured chondrocytes or freshly harvested synovial membrane and cartilage tissues. Galectin-1 and -3 expression did not vary significantly with passage from passage 1 to passage 3 BMSCs (data not shown). Galectin-1 expression was elevated in cultured articular cells as compared to freshly harvested joint tissues, with galectin-1 significantly increased in cultured synoviocytes as compared to synovial membrane tissue (*p* = 0.004) and cultured chondrocytes as compared to articular cartilage tissue (*p* < 0.0001).

### Cytokine-influenced BMSC galectin gene and protein expression

**Fig. 3:**
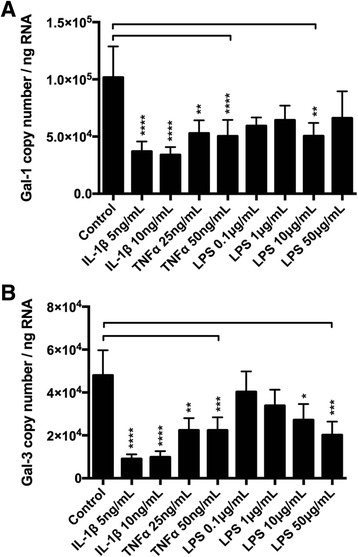
Inflammation decreases galectin gene expression in BMSCs. Galectin-1 and galectin-3 expression levels are reduced following exposure to pro-inflammatory stimuli. **a** Galectin-1 and (**b**) galectin-3 transcription levels (copy number/ng RNA) in equine passage 3 BMSCs 20 h after stimulation with recombinant equine interleukin-1 beta (*IL-1β*), recombinant equine tumor necrosis factor alpha (*TNF-α*), or lipopolysaccharide (*LPS*). Data are presented as mean ± SE of three independent experiments in which three different BMSC donors were analyzed in duplicate. Statistical analysis is performed using Friedman’s test with post hoc comparisons as described by Gibbons and Chakraborti (p. 459, equation 2.13) [52] using a Bonferroni correction for multiple comparisons (*p < 0.05, **p < 0.01, ***p < 0.001, ****p < 0.0001)

The pro-inflammatory cytokines IL-1β and TNF-α decreased both galectin-1 and galectin-3 mRNA expression in equine BMSCs. IL-1β at 5–10 ng/mL decreased BMSC galectin-1 and galectin-3 mRNA expression more than 3-fold at 20 h post-exposure (Fig. 3a, b). Exposure to 25–50 ng/mL of TNF-α also decreased galectin-1 and galectin-3 mRNA expression, but to a lesser extent than IL-1β (Fig. 3a, b). The effect of LPS treatment was less pronounced. LPS did not significantly decrease galectin-1 mRNA expression at 20 h (Fig. 3a), and only the highest LPS dose of 50 μg/mL resulted in a decrease in galectin-3 mRNA expression (*p* = 0.05, Fig. 3b).

**Fig. 4:**
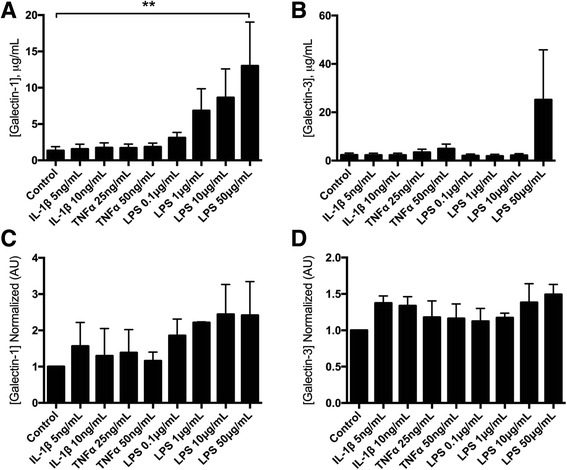
Secreted and intracellular/membrane-bound galectins in BMSCs. **a** Galectin-1 and (**b**) galectin-3 secretion (μg/mL) measured in the supernatants of cytokine- or LPS-treated equine passage 3 BMSCs 20 h following treatment. **c** Galectin-1 and (**d**) galectin-3 concentrations measured in cell lysates from cytokine- or lipopolysaccharide (*LPS*)-treated equine passage 3 BMSCs 20 h following treatment. For all graphs, data are presented as mean ± SE of three independent experiments in which three different BMSC donors were analyzed in duplicate. Statistical analysis is performed using Friedman’s test with post hoc comparisons as described by Gibbons and Chakraborti (p. 459, equation 2.13) [52] using a Bonferroni correction for multiple comparisons. Galectin-1 concentrations were increased only in supernatants from MSCs exposed to the 50 μg/mL dose of LPS (**p < 0.01). No significant differences were observed for galectin-3 supernatants or galectin-1/galectin-3 cell lysates. *IL-1β* interleukin-1 beta; *TNF-α* tumor necrosis factor alpha

Although galectin mRNA expression decreased following exposure to both IL-1β and TNF-α, no changes were observed in galectin protein concentrations in media supernatants upon treatment with either cytokine at 20 h post-exposure (Fig. 4). Immunoblotting of BMSC lysates similarly did not reveal any quantitative differences in intracellular or membrane-bound galectins-1 and 3 (Fig. 4). LPS treatment at 50 μg/mL resulted in an increase in galectin-1 protein concentrations in media supernatants 20 h post-exposure, whereas galectin-3 concentrations in media supernatants did not significantly differ from controls (Fig. 4).

### BMSC adhesion with focal adhesion markers

**Fig. 5:**
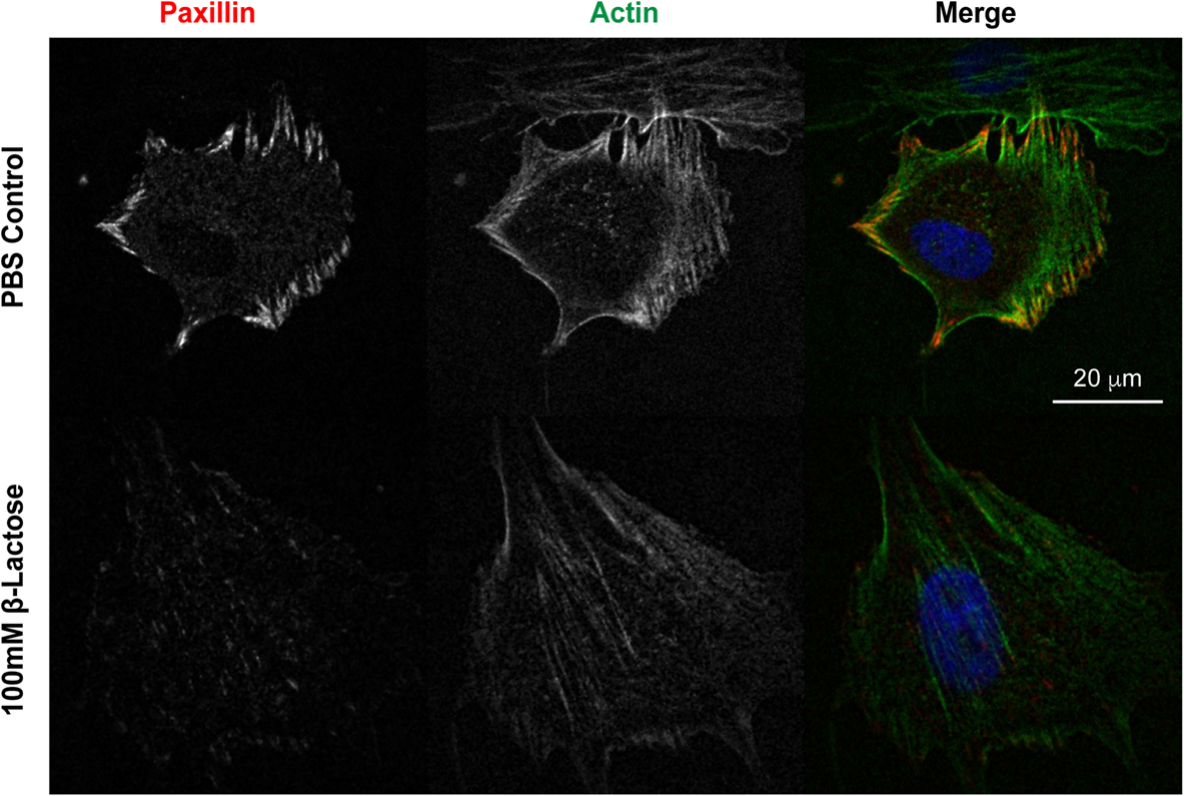
Galectins promote focal adhesion formation. Equine passage 4 BMSCs plated on fibronectin-coated glass substrates following nucleofection with the focal adhesion marker paxillin mEmerald and staining for actin (phalloidin Alexa647) and nuclei (DAPI). Distinct focal adhesion structures are present within BMSCs plated in control media but not in media with 100 mM β-lactose

When plated on fibronectin-coated glass substrates, equine passage 4 BMSCs nucleofected with paxillin demonstrated distinct focal adhesions at the cell periphery (Fig. 5). In addition, focal adhesions were associated with actin stress fibers, indicating mature, mechanically stable adhesive structures. The addition of 100 mM β-lactose inhibited adhesion complex assembly, suggesting that galectins play a role in BMSC adhesion, at least in part through integrin-mediated mechanisms.

### BMSC cell migration

**Fig. 6:**
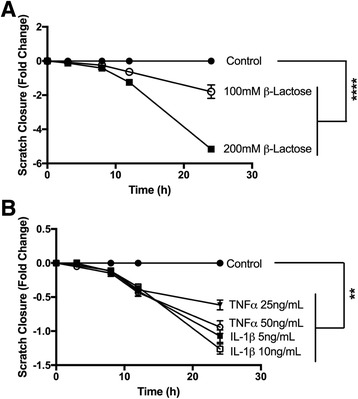
BMSC migration data. Scratch closure by equine passage 3 BMSCs, expressed as fold change in a 500 μm cell-free gap as compared to the control treatment for each time point. Data are presented as mean ± SE from three independent experiments performed in duplicate. **a** Treatment with 100 mM β-lactose and 200 mM β-lactose significantly abrogated BMSC motility as compared to control (*p* < 0.0001, Dunnett’s test). **b** Treatment with the pro-inflammatory cytokines interleukin-1 beta (*IL-1β*) or tumor necrosis factor alpha (*TNF-α*) decreased BMSC motility as compared to control (*p* < 0.01, Dunnett’s test)

Regression analysis of migration data revealed a dose-dependent reduction in BMSC motility in the presence of β-lactose (Fig. 6a, Additional file 2). IL-1β (5 ng/mL, 10 ng/mL) and TNF-α (25 ng/mL, 50 ng/mL) both significantly decreased BMSC motility relative to untreated controls (Fig. 6b, Additional file 2).

## Discussion

Equine BMSCs constitutively express elevated levels of galectin-1 relative to other articular cell types. Galectin-1 and galectin-3 have both been previously identified in the proteome of fibroblast-like synoviocytes from patients with rheumatoid arthritis [53]. Although galectin gene expression has not been extensively studied in articular cartilage, galectin-3 gene expression was increased more than 2-fold in human OA cartilage as compared to normal cartilage [34]. In order to determine how galectin expression in equine BMSCs compared to galectin expression in other articular tissues, galectin-1 and -3 mRNA was quantified in synovial membrane and articular cartilage tissues obtained from the carpal joints of horses without evidence of OA. Furthermore, in order to evaluate the effect of tissue culture expansion, galectin expression was measured in freshly harvested articular tissues, in addition to cultured synoviocytes and chondrocytes. Although culturing cells increased galectin-1 expression in both synoviocytes and chondrocytes, galectin-3 mRNA expression was unchanged. BMSC galectin-1 expression remained significantly higher than either cultured synoviocytes or chondrocytes.

Galectin-1 and -3 expression was lowest in healthy equine articular cartilage tissue and cultured chondrocytes. Galectins-1, -3 and -8 have been documented at both gene and protein levels in cultured human OA chondrocytes, and immunohistochemical staining has revealed elevation of galectin-1 in areas of severe cartilage degeneration [35] and elevation of galectin-3 in OA chondrons [54]. Altered glycosylation in human OA chondrocytes induced by IL-1β and TNF-α has led authors to speculate that galectin function may be altered under inflammatory arthritis conditions [55]. Access to healthy human articular cartilage is limited by tissue availability; therefore, findings in human tissues may be skewed toward a disease or OA phenotype. Furthermore, changes in galectin gene and protein expression in human articular cartilage are not necessarily paralleled by synovial membrane or synovial fluid changes.

For example, although human articular chondrocytes appear to increase galectin expression under inflammatory OA conditions, synovial fluid galectin-1 concentrations are decreased in human OA [29], juvenile idiopathic arthritis [30, 31] and rheumatoid arthritis [32]. Furthermore, elevated synovial fluid galectin-1 concentrations have been shown to protect against the development OA. Evidence supporting the protective role of galectin-1 in OA can be extrapolated from murine models where galectin-1 gene therapy and recombinant galectin-1 intra-articular administration ameliorated adjuvant-induced arthritis [38, 56]. Gal-1/-1 knockout mice show exacerbation of post-traumatic arthritis [37]. The mechanistic roles of galectin-1 and galectin-3 in human and equine arthritis are less well understood; however, increased synovial fluid galectin-1 is thought to be protective as galectin-1 is immunomodulatory in other chronic inflammatory diseases [27, 28].

We observed constitutive galectin-1 and -3 mRNA expression in equine synovial fibroblast cultures; however, equine BMSCs expressed significantly higher levels of galectin-1 and increased galectin-1:galectin-3 ratios as compared to other articular-derived cells. BMSCs had an elevated galectin-1:galectin-3 ratio (3.77) as compared to synoviocytes and chondrocytes (0.62 and 0.34, respectively) and synovial membrane and articular cartilage tissue (0.65 and 0.02, respectively). Elevated expression of galectin-1 relative to galectin-3 may contribute to the beneficial immunomodulatory properties of BMSCs based on prior studies suggesting that increased galectin-1 and decreased galectin-3 synovial fluid concentrations are anti-inflammatory and ameliorate the progression of OA [31, 37, 38, 57]. Furthermore, galectin-1 has been shown to confer immunomodulatory properties of MSCs through inhibition of T cells [15, 17, 21] and attenuation of pro-inflammatory cytokine expression [58].

In the presence of the inflammatory cytokines IL-1β and TNF-α, equine BMSCs downregulated expression of both galectin-1 and galectin-3 mRNA. Although downregulation of galectin-1 might be detrimental to the immunomodulatory properties of intra-articularly injected BMSCs, decreases in galectin gene expression were not paralleled by decreases in protein expression in BMSC lysates or media supernatants. One possibility for the discrepancy between the gene and protein expression results is that galectin mRNA levels do not predict protein levels due to variation in rates of translation, protein stability, and/or protein degradation. Several studies have demonstrated that a gene’s mRNA level is not necessarily a good predictor of protein level, with correlation coefficients between gene and protein expression rarely exceeding 0.43 [59, 60]. Another possibility is that 20–30 h was not a sufficient period of time to observe protein-level changes in cultured BMSCs. A previous report revealed that TNF-α reduced galectin-3 protein expression as measured by flow cytometry in human OA and rheumatoid arthritis synovial fibroblasts 18 hours following exposure to TNF-α [61]. To our knowledge, MSC galectin-1 and -3 protein expression has not been previously quantified by ELISA or immunoblotting in response to IL-1β and TNF-α. Future studies may need to evaluate MSC galectin protein expression at longer intervals following inflammatory cytokine exposure to confirm that galectin protein expression is not changing significantly. In addition, although the concentrations of pro-inflammatory cytokines tested were similar to those used in prior studies [43, 62], it is likely that the concentrations used in this study are much higher than those found in naturally occurring equine OA [50].

Confocal imaging of equine BMSCs nucleofected with the focal adhesion protein paxillin-mEmerald revealed distinct adhesion complexes on fibronectin-coated glass substrates. Galectins have been shown to interact with integrins [63–66], and integrins play a significant role in MSC adhesion, spreading and motility [67, 68]. Several adhesion-related molecules have been detected in human MSCs, including integrin subunits α4, α5, β1, ανβ3, ανβ5, ICAM-1 and CD44H [69]. Galectins bind directly to integrins, promoting both integrin clustering [65] and signaling activity [66]. Integrins are thought to play a major role in galectin-3-mediated regulation of cell adhesion [63]. Galectin-3 can bind to integrins such as α_1_β_1_ [70], and galectins can enhance the mechanical strength of adhesive bonds between integrins and extracellular matrix proteins, such as collagen-I and -IV [64]. Interestingly, inhibition of BMSC galectin binding with 100 mM β-lactose nearly abolished focal adhesion structures and decreased MSC adhesion and spreading. This suggests that galectins play an important role in equine BMSC adhesion to extracellular matrices.

Equine BMSC motility was markedly downregulated in the presence of the pan-galectin inhibitor β-lactose, suggesting that galectins may play a critical role in stem cell migration. Galectin-3 promotes wound re-epithelialization in corneal, intestinal and skin wounds [71], and galectin-1 accelerates skin wound healing [72]. Galectin-1 enhances migration of human monocyte-derived dendritic cells through extracellular matrices [73] and stimulates motility of human umbilical cord blood-derived MSCs via downregulation of Smad2/3 and upregulation of NF-kB [19]. Conversely, priming of BMSCs with pro-inflammatory cytokines (IL-1β and TNF-α) decreased BMSC motility. These results are consistent with a prior study in which equine BMSCs exposed to TNF-α and IFN-ϒ significantly downregulated migration-related genes such as the chemokine receptor *CXCR4* [43]. Further studies would be needed to determine the effects of pro-inflammatory cytokine priming on BMSC motility in vivo and in response to physiologic concentrations of inflammatory cytokines present in OA synovial fluid.

## Conclusions

Equine BMSCs express high levels of galectin-1 mRNA relative to other articular cell types; however, BMSC galectin mRNA expression is diminished in the presence of pro-inflammatory cytokines prevalent in osteoarthritis (IL-1β and TNF-α). Under the conditions tested in this study, decreased galectin mRNA expression was not paralleled by decreases in galectin protein expression. Therefore, intra-articular administration of BMSCs may be a viable method to achieve therapeutic levels of intra-articular galectin-1, even in the presence of joint inflammation. Pro-inflammatory cytokines and β-lactose, a pan-galectin inhibitor, impaired BMSC motility. Further investigation into the effects of joint inflammation on BMSC function and the potential therapeutic effects of BMSC galectin expression on joint inflammation and osteoarthritis is warranted.

## Additional files

Additional file 1: Antibody dot blots for recombinant equine galectins. Recombinant equine galectins-1 and -3 probed with antibodies used for custom galectin ELISAs, including: (A) (R&D Systems, Minneapolis, MN, USA) goat α-mouse Gal-1 pAb, AF1245, (B) (Santa Cruz Biotechnology, Dallas, TX, USA) goat α-human Gal-3 pAb, sc-19280, and (C) (R&D Systems, Minneapolis, MN, USA) biotinylated goat α-mouse Gal-3 pAb, BAF1197. Five μL of recombinant equine (reGal) or human (rhGal) galectin or BSA was pipetted onto a nitrocellulose membrane at concentrations of 500 μg/mL (1), 100 μg/mL (1:5) and 20 μg/mL (1:25, top to bottom). Following blocking in milk + 2% BSA, proteins were immunodetected. (TIF 1538 kb)

Additional file 2: Regression analysis and least squares means for BMSC migration data. Results of BMSC migration data reported as mean cell-free gap within a 500 μm scratch (normalized to time 0 h), presented as least squares geometric means and standard error (SE). Effects included time, treatment and equine BMSC primary cell line. The intercept is excluded for clarity. (PDF 34 kb)

## Abbreviations

BMSC: Bone marrow-derived mesenchymal stromal cell
IFN-ϒ: Interferon gamma
IL-1β: Interleukin-1 beta
LPS: Lipopolysaccharide
MSC: Mesenchymal stromal cell
OA: Osteoarthritis
TNF-α: Tumor necrosis factor alpha
