# Supplementary figures and images for: Galectin-1 and galectin-3 expression in equine mesenchymal stromal cells (MSCs), synovial fibroblasts and chondrocytes, and the effect of inflammation on MSC motility
Source: Stem Cell Res Ther. 2017 Nov 2;8:243. doi: 10.1186/s13287-017-0691-2 (PMC5667510; doi:10.1186/s13287-017-0691-2)

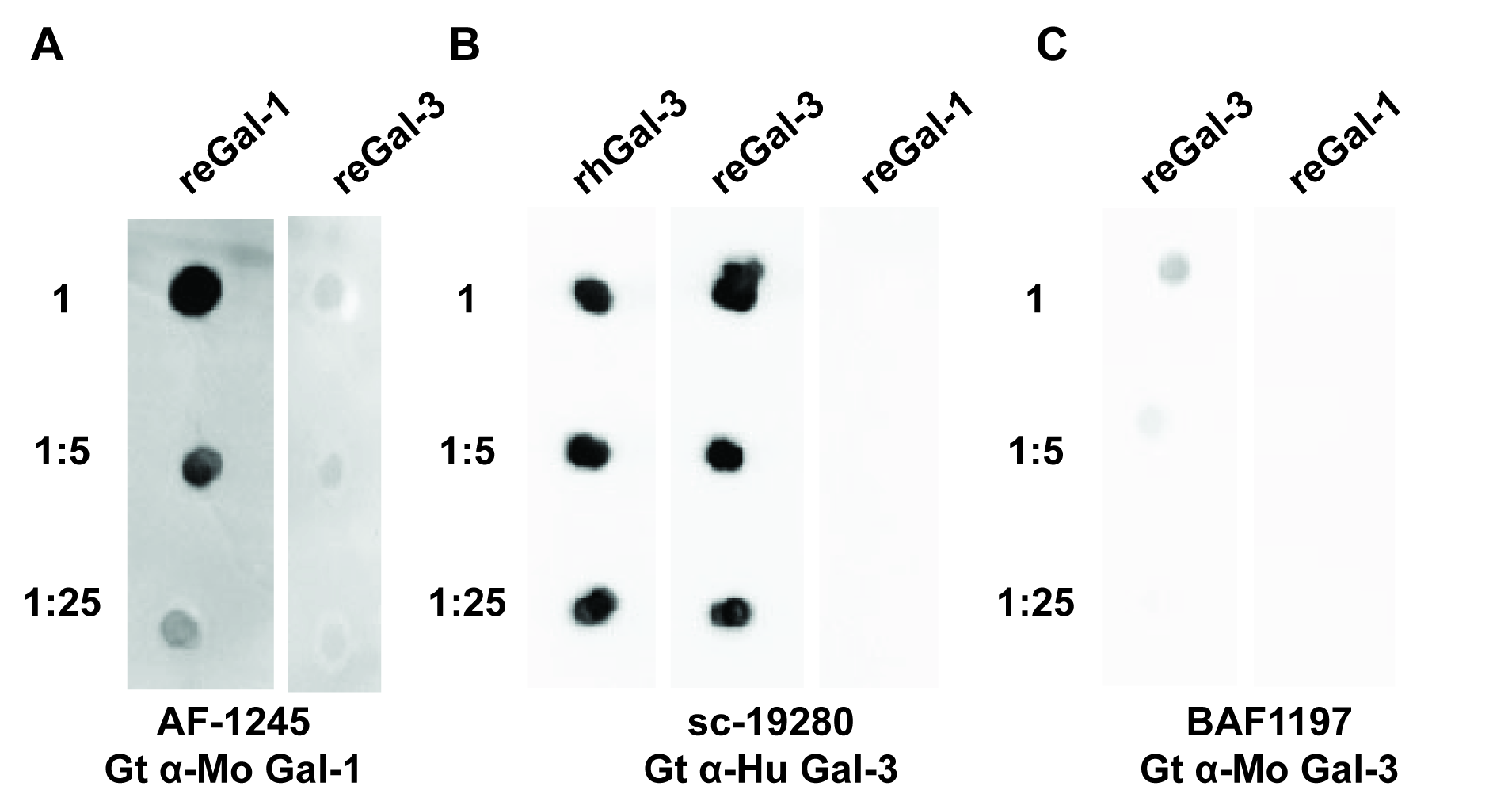

Supplement: Supplementary file 1 — Antibody dot blots for recombinant equine galectins. Recombinant equine galectins-1 and -3 probed with antibodies used for custom galectin ELISAs, including: (A) (R&D Systems, Minneapolis, MN, USA) goat α-mouse Gal-1 pAb, AF1245, (B) (Santa Cruz Biotechnology, Dallas, TX, USA) goat α-human Gal-3 pAb, sc-19280, and (C) (R&D Systems, Minneapolis, MN, USA) biotinylated goat α-mouse Gal-3 pAb, BAF1197. Five μL of recombinant equine (reGal) or human (rhGal) galectin or BSA was pipetted onto a nitrocellulose membrane at concentrations of 500 μg/mL (1), 100 μg/mL (1:5) and 20 μg/mL (1:25, top to bottom). Following blocking in milk + 2% BSA, proteins were immunodetected. (TIF 1538 kb) [file 13287_2017_691_MOESM1_ESM.tif]
