# Supplementary material for: Galectin-1 and galectin-3 expression in equine mesenchymal stromal cells (MSCs), synovial fibroblasts and chondrocytes, and the effect of inflammation on MSC motility
Source: Stem Cell Res Ther. 2017 Nov 2;8:243. doi: 10.1186/s13287-017-0691-2 (PMC5667510; doi:10.1186/s13287-017-0691-2)
Supplement: Supplementary file 2 — Regression analysis and least squares means for BMSC migration data. Results of BMSC migration data reported as mean cell-free gap within a 500 μm scratch (normalized to time 0 h), presented as least squares geometric means and standard error (SE). Effects included time, treatment and equine BMSC primary cell line. The intercept is excluded for clarity. (PDF 34 kb) [file 13287_2017_691_MOESM2_ESM.pdf]

Additional File 2. Results of BMSC migration data reported as mean cell-free gap within a 500 μm scratch (normalized to time 0 h), presented as least squares geometric means and standard error (s.e.). Effects included time, treatment and equine BMSC primary cell line. The intercept is excluded for clarity.

Effect Tests

**Source**	**DF**	**F Ratio**	**Prob > F**	**Fit**
Time	5	716.09	<0.0001	R^2: 0.907
Treatment	6	65.46	<0.0001	R^2 Adj: 0.904
Cell Line	2	57.25	<0.0001	

**Least Squares Means Table**		***P*-value**
**Time (h)**	**LSM (± SE)**	**Prob > F**
		<0.0001
0	1.014 (0.013)[a]	
3	0.930 (0.013)[b]	
8	0.808 (0.013)[c]	
12	0.605 (0.013)[d]	
24	0.325 (0.013)[e]	
48	0.126 (0.013)[f]	
**Treatment**	**LSM (± SE)**	**Prob > F**
		<0.0001
Control	0.899 (0.019)	
TNFα 25ng/mL	0.963 (0.019)[**]	
TNFα 50ng/mL	0.986 (0.019)[****]	
IL-1β 5ng/mL	0.979 (0.019)[****]	
IL-1β 10ng/mL	0.977 (0.019)[****]	
100mM β-Lactose	1.036 (0.019)[****]	
200mM β-Lactose	1.257 (0.019)[****]	
**Horse Cell Line**	**LSM (± SE)**	**Prob > F**
		<0.0001
Cell Line 1	1.012 (0.015)[a]	
Cell Line 2	0.945 (0.015)[b]	
Cell Line 3	1.086 (0.015)[c]	

[a,b,c,d,e,f] Means with different superscript letters differ at level $p < 0.05$ (Tukey's *post hoc* test).
[**,****] Means with different superscript asterisks differ at level * $p < 0.01$, ** $p < 0.0001$ (Dunnett's test).
